# Direct Evaluation of L-DOPA Actions on Neuronal Activity of Parkinsonian Tissue *In Vitro*

**DOI:** 10.1155/2013/519184

**Published:** 2013-09-17

**Authors:** Víctor Plata, Mariana Duhne, Jesús E. Pérez-Ortega, Janet Barroso-Flores, Elvira Galarraga, José Bargas

**Affiliations:** División de Neurociencias, Instituto de Fisiología Celular, Universidad Nacional Autónoma de México (UNAM), 04510 México City, DF, Mexico

## Abstract

Physiological and biochemical experiments *in vivo* and *in vitro* have explored striatal receptor signaling and neuronal excitability to posit pathophysiological models of Parkinson's disease. However, when therapeutic approaches, such as dopamine agonists, need to be evaluated, behavioral tests using animal models of Parkinson's disease are employed. To our knowledge, recordings of population neuronal activity *in vitro* to assess anti-Parkinsonian drugs and the correlation of circuit dynamics with disease state have only recently been attempted. We have shown that Parkinsonian pathological activity of neuronal striatal circuits can be characterized in *in vitro* cerebral tissue. Here, we show that calcium imaging techniques, capable of recording dozens of neurons simultaneously with single-cell resolution, can be extended to assess the action of therapeutic drugs. We used L-DOPA as a prototypical anti-Parkinsonian drug to show the efficiency of this proposed bioassay. In a rodent model of early Parkinson's disease, Parkinsonian neuronal activity can be returned to control levels by the bath addition of L-DOPA in a reversible way. This result raises the possibility to use calcium imaging techniques to measure, quantitatively, the actions of anti-Parkinsonian drugs over time and to obtain correlations with disease evolution and behavior.

## 1. Introduction

Idiopathic Parkinson's disease is a progressive incapacitating movement disorder whose treatment remains unsatisfactory. Behavioral tests are commonly used in Parkinsonian animal models to assess experimental therapeutic approaches and drugs intended to be helpful in Parkinsonian patients [1]. Nevertheless, after half a century, L-DOPA remains the treatment of choice and the “gold standard” [2]. However, experimental paradigms to efficiently assess new anti-Parkinsonian drugs and treatments using *in vitro* brain tissue, during neuronal activity, are lacking.

Recently, we used calcium imaging techniques to record dozens of neurons simultaneously with single-cell resolution to prove that the activity of the striatal microcircuit recorded *in vitro* during dopamine (DA) depletion is radically different than that recorded in the controls [3]. As previously reported, the Parkinsonian circuit had increased synaptic and neuronal activity [4–10] and was particularly engaged into a dominant network state [3] due to increased neuronal synchronization [3, 11–13]. The action of a DA D_1_-receptor agonist in this circuit dissolved the dominant state but did not reverse the enhanced activity [3]. In contrast, it has been shown that the use of DA D_2_-receptor agonists appears to better restore the balance between direct and indirect pathways activity [14], suggesting that D_2_-receptor agonists may be better therapeutic agents. However, this latter action may be indirect, since inhibitory connections are stronger from indirect to direct pathway neurons [15, 16] and D_2_-receptor agonists may also inhibit these synapses [17], thus, suppressing their braking effect on direct neurons excitability. In addition, previous work has also stated that the synergistic activation of both D_1_- and D_2_-receptor agonists may be necessary to have a better amelioration of signs and to delay dyskinesias [18–21], a good reason why L-DOPA remains as the “gold standard” [2].

Due to this controversy, we asked whether an *in vitro* bioassay can be designed to test the actions of drugs directly onto neuronal activity. Therefore, the goals of the present report are as follows: first, to test the actions of L-DOPA directly on brain tissue maintained *in vitro* (corticostriatal brain slice preparation); second, to observe the actions of L-DOPA on the simultaneous activity of dozens of neurons in the DA-depleted striatal microcircuit. In particular, we wanted to see L-DOPA actions at a stage considered as early Parkinsonian and determine whether it can dissolve the dominant network state [3]. Finally, we want to see, in the present preparation, whether a measure of global population activity may be enough to compare different drugs and open the path to perform, in the future, concentration-response functions.

The present work shows that, in the early Parkinsonian state, L-DOPA restores neuronal activity to control levels. This action was readily reversible. Therefore, the present prototypical experimental design is open to develop more sophisticated network analyses to expose finer differences between therapeutic agents [22, 23], since single-cell resolution is maintained. To conclude, the present preparation deserves further exploration in order to validate a bioassay to investigate and compare the actions of anti-Parkinsonian agents quantitatively.

## 2. Material and Methods

### 2.1. Slice Preparation

Male mice or rats were housed in clear plastic cages and maintained on a stable 12 : 12 hours dark/light cycle at room temperature (22°C), with food and water ad libitum in our Animal House. The number of animals used in the experimental samples was the minimal possible to attain statistical significance. All the procedures followed the National University of Mexico guidelines, the Guide for the Care and Use of Laboratory Animals (National Institutes of Health), in accordance with the EC Directive 86/609/EEC for animal experiments. The slice preparation and its analysis have been described before [3, 20, 22]. Briefly, corticostriatal slices (250 *μ*m in thickness) were obtained either from control or 6-hydroxydopamine (6-OHDA) treated rodents (mice or rats, postnatal day 23–29).

Because experiments using 6-OHDA treated animals were done mostly in mice, the timing of the protocol in these animals is reported: lesion with 6-OHDA was done at day 21, turning behavior to check the hemi-Parkinsonian model was done at day 24, and recording of slices with calcium imaging was done at day 26 or later. Slices were cut in ice-cold bath saline (4°C) containing the following (in mM): 123 NaCl, 3.5 KCl, 1 MgCl_2_, 1 CaCl_2_, 26 NaHCO_3_, and 11 glucose (25°C; saturated with 95% O_2_ and 5% CO_2_; pH = 7.4; 298 mOsm/L). Slices were then transferred to saline at room temperature (21–25°C) where they remained for at least 1 h before recording.

### 2.2. The 6-OHDA Hemi-Parkinsonian Model

The method to produce the 6-OHDA rodent model of hemi-Parkinsonism, in rats and mice, has been described extensively [3, 24]. Degree of lesion in animals was tested by both tyrosine hydroxylase (TH) immunocytochemistry and turning behavior [24]. The striatum from the injured side was tested one week after the behavioral test.

### 2.3. Calcium Imaging

Calcium imaging techniques to record, indirectly, the electrical activity of dozens of neurons simultaneously during control conditions and during DA depletion have also been extensively described [3, 20, 22–24]. In this work, we employed 10 *μ*M fluo-8 AM (Tef Labs, Austin, TX; with 0.1% DMSO) and an upright microscope with 10x water immersion objective (E600FN Eclipse, Nikon, Melville, NY). A series of images were acquired with a cooled CCD camera (SenSys 1401E, Roper Scientific, Tucson, AZ) at 100–250 ms/frame.

Active neurons within the field of view were automatically selected by a program written in LabView (National Ins. Mexico) which also processed the sequence of images. The changes of fluo-8 fluorescence originated from neuronal discharge [22–24] eliciting Ca^2+^ signals as (*F*_*i*_ − *F*_*o*_)/*F*_*o*_, where *F*_*i*_ is fluorescence intensity at any frame and *F*_*o*_ is resting fluorescence. Duration of neuronal discharge was approximated based on the first time derivative of these Ca^2+^ signals [22]. Statistical significance used a threshold value given by 2.5 times the standard deviation of the noise. In this way, the neuronal activity over time was graphed in raster plots, where each line in the *y*-axis represents the activity of a single neuron and the *x*-axis represents time in minutes. A histogram shows the summed activity of all neurons (as a field recording) except that each neuron composing a peak of synchronization can be identified. Here, the histogram was used to detect episodes with spontaneous synchronization of several neurons. A horizontal bar indicates the time when L-DOPA was added to the bath saline.

### 2.4. Statistical Methods

Most statistical methods used have been previously described [22, 23]. To determine the statistical significance of a set of coactive neurons (neuronal vectors), Monte Carlo simulations with 10,000 replications were performed. To analyze the dynamics of the neuronal microcircuit, we employed network states or significant peaks of coactive neurons [22, 23]. A similarity index was used to determine if these peaks of synchronization contained the same or different neurons. A method for dimensional reduction, locally linear embedding (LLE), was used to project the neuronal vectors in a plane and, thus, follow the dynamics of the circuit [3, 22–24]. To compare global neuronal activity over a given time, we plotted both the cumulative distribution of all cell activity and the cumulative recruitment of neurons. The rates of these cumulative relationships were approximated with *ad hoc* linear regressions. Their average rates of change (slopes) ± their estimation errors were compared for significant differences with nonpaired Student's *t* tests, experiment by experiment. Average significance is reported. In addition, for sample comparisons of these parameters, we used Wilcoxon's *T* statistic for paired samples and Kruskal-Wallis statistic for nonpaired samples.

### 2.5. Chemicals

3,4-Dihydroxy-L-phenylalanine (L-DOPA), 6-hydroxy-dopamine hydrobromide (6-OHDA), and dimethyl sulfoxide (DMSO) were obtained from Sigma-Aldrich (Mexico), Fluo-8-AM and Fluo-8-sodium salt were obtained from AAT Bioquest (Sunnyvale, CA), and ketamine hydrochloride and xylazine hydrochloride were obtained from PiSA (Mexico).

## 3. Results and Discussion

**Figure 1:**
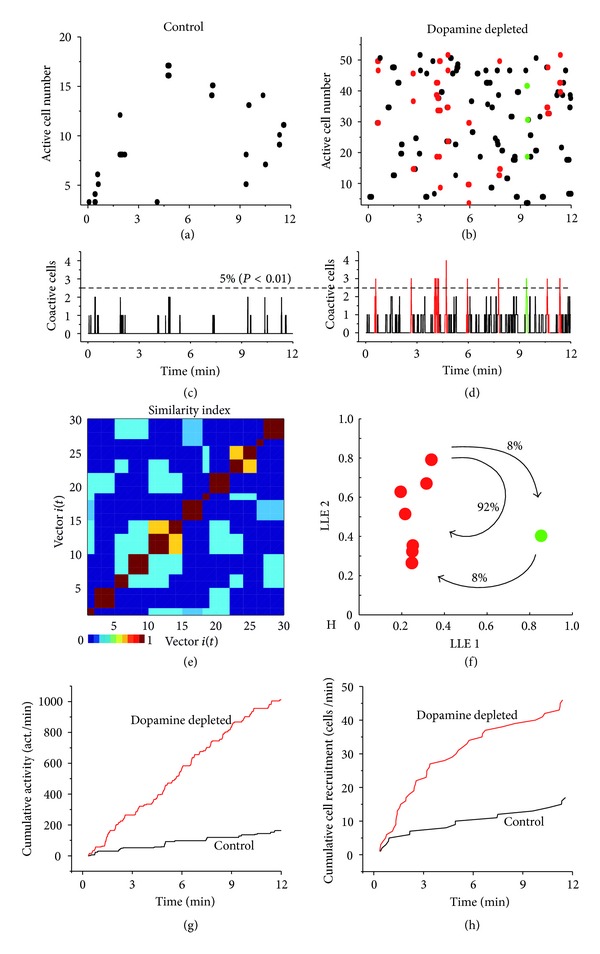
Control and dopamine-depleted striatal microcircuits. (a), (b) Raster plots showing spontaneous neuronal activity in striatal microcircuits in control (a) and after dopamine (DA) depletion (b). Note, increased activity in the DA-depleted circuit. (c), (d) Histograms of summed activity (columns) shown in raster plots frame by frame. Only the DA-depleted microcircuit had significant peaks of coactive neurons: peaks of synchronization of the same color indicate that similar neurons discharged in those instances. (e) Similarity indices of all neuronal vectors representing network states as a function of time in the DA-depleted circuit. (f) Multidimensional reduction of vectors representing network states using locally linear embedding (LLE). Note that one neuronal vector (red: projections of the peaks of synchronization above) activates more frequently creating a dominant network state that absorbs most active neurons. (g), (h) Cumulative activity and cumulative cell recruitment in both control (black traces) and DA-depleted circuit (red traces).

As shown in previous work, the striatal circuit appears almost silent in control conditions, with very little activity (Figure 1(a): each point represents the discharge of a neuron and each line denotes the activity of a neuron along time) [3, 8, 10, 11]. In contrast, after DA depletion neuronal activity in the striatal circuit is enhanced [3–10] (Figure 1(b)). The histogram of summed activity does not show significant peaks of synchronization in the control (Figure 1(c)) but exhibits spontaneous and significant peaks of neuronal synchronization (network states in color) in the DA-depleted tissue. This finding is in agreement with field potential waves or oscillations recorded electrophysiologically [3–13] (*n* = 6; Figure 1(d)).

The color of synchronization peaks (column neuronal vectors) indicates that the same active neurons make up the peaks (confirmed by the similarity index of the Parkinsonian activity; Figure 1(e): which shows abundance and repetitive activation of a similar patterned mosaic). Dimensional reduction using LLE [22] confirms that most network states contain similar neurons (red), suggesting that circuit dynamics is reduced to the frequent recurrent activity of a dominant network state [3] (red; Figure 1(f)). Figures 1(g) and 1(h) illustrate ways to measure global neuronal activity over a time span to compare control and DA-depleted circuits. Cumulative activity indicates consecutive addition of all bars (active neurons) of activity histograms (Figures 1(c) and 1(d) over time (Figure 1(g)) in both control (black traces) and the DA-depleted circuit (red traces). The rate of change (tendencies) of cumulative activities can be approximated with *ad hoc* fitting of straight lines where the rates of cumulative activity are the slopes of the lines ± their standard estimation errors (95% confidence). In control (Figure 1(g)), rate of cumulative activity was significantly larger for DA-depleted than control tissue (mean rate ± mean estimation error): 35 ± 0.12 act./min in the controls, while it was 105 ± 0.28 act./min in the DA-depleted tissue (*n* = 6; *P* < 0.001) [3]. Cumulative cell recruitment shows the addition of new neurons activated over time (Figures 1(a) and 1(b) in both control (black traces) and the DA-depleted circuit (red traces; Figure 1(h)). In controls (Figure 1(h)), rate of neuronal recruitment over time was significantly larger for DA-depleted than control tissue (mean rate ± mean estimation error): 4 ± 0.26 cells/min in the controls, while it was 9 ± 0.31 cells/min in the dopamine-depleted tissue (*n* = 6; *P* < 0.0025). 

In summary, it is shown that prominent attributes previously reported for the DA-depleted circuit as compared to the controls were fulfilled in the present sample of *in vitro* experiments. There was more activity in the DA-depleted tissue than in the control [3–10], this increased activity was characterized by the spontaneous synchronization of neurons, and because these neurons conformed similar peaks of synchronization, we conclude that they are mostly caused by the recurrent activity of the same network state [3].

**Figure 2:**
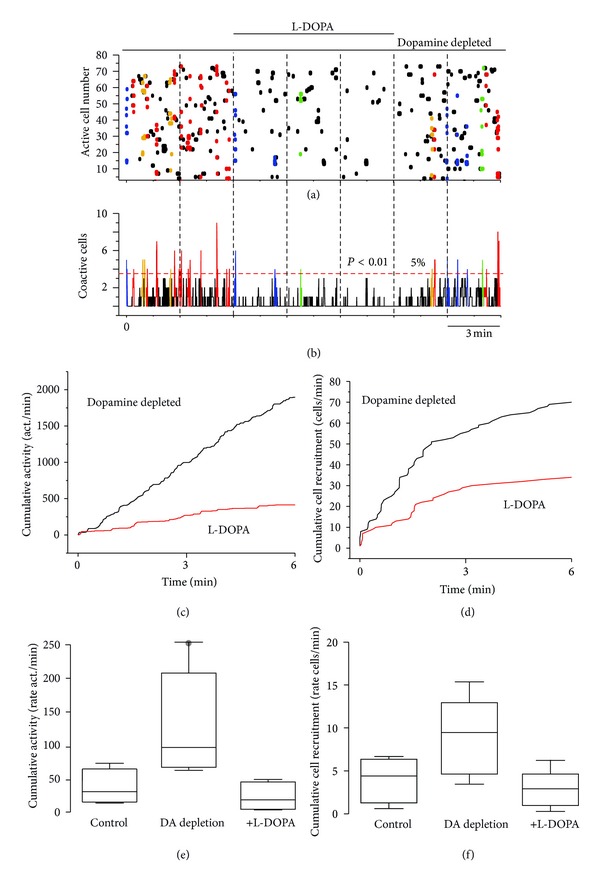
Actions of L-DOPA on DA-depleted striatal microcircuit. (a) Raster plot showing neuronal activity before and during the administration of 1 *μ*M L-DOPA into the bath saline (black bar). Note that neuronal activity is reduced during L-DOPA. L-DOPA actions are reversible. (b) Summed neuronal activity histogram: abundant peaks of neurons firing in synchrony are present before and after but not during L-DOPA present in the bath saline. (c), (d) Cumulative activity and cumulative cell recruitment in the DA-depleted microcircuit both before (black traces) and during L-DOPA administration (red traces). (e), (f) Tukey box plots showing samples distributions of cumulative activity and cumulative cell recruitment, respectively. Neuronal activity is significantly different when one compares DA-depletion versus either control (*P* < 0.05) or L-DOPA (*P* < 0.028). Difference between control and L-DOPA samples is not significant.

Figure 2(a) shows the activity of over 70 neurons in DA-depleted tissue, and the histogram in Figure 2(b) shows the summed activity of all neurons [22]. Colors indicate neuronal column vectors or peaks of synchronization as indicated by the similarity index. Because the animals are hemi-Parkinsonian after a lesion with 6-OHDA (see Section 2), first two frames show that the circuit activity is higher than in control animals (DA depleted, cf. Figure 1(a)). This increased pathological activity is the control in the present experiment. Next three frames in Figure 1(a) show the activity of the DA-depleted circuit during the addition of 1 *μ*M L-DOPA to the bath saline (bar). Note that L-DOPA gradually eliminated peaks of coactive cells and virtually restores control resting activity. Washing off L-DOPA restored Parkinsonian activity (last two frames), showing that actions of the drug were reversible.

Note that the rate of cumulative activity (Figure 2(c)) decreased after addition of 1 *μ*M L-DOPA to the bath saline, from (mean rate ± mean estimation error) 105 ± 0.28 act./min (DA-depleted tissue) to 24 ± 0.15 (*n* = 6; *P* < 0.001). The rate of cell recruitment over time also decreased from 9 ± 0.31 to 3 ± 0.25 (*P* < 0.001). The changes are clear at a glance and samples are summarized with box Tukey distributions (medians and percentiles) in Figures 2(e) and 2(f). The DA-depleted sample and the sample with L-DOPA administration employed the same tissue (since striatal tissue from 6-OHDA injured animals was used for controls and the test sample used the same tissue in the presence of L-DOPA). Therefore, parameters significance between these samples was tested with a Wilcoxon's *T* paired statistic (*P* < 0.028). Control sample was taken from different animals, and there were no significant differences between its parameters and those from the L-DOPA sample, suggesting that during early stages of the Parkinsonian state, L-DOPA is capable of restoring neuronal activity to levels that are undistinguishable from those in the controls. However, there were significant differences between control and DA-depleted samples (Kruskal-Wallis, *P* < 0.05).

It is shown that calcium imaging of neuronal populations allows direct observation and measurement of the actions of anti-Parkinsonian drugs on the dopamine-depleted striatal microcircuit. We used the gold standard of anti-Parkinsonian therapeutics: L-DOPA [2], in order to compare its actions with other drugs tested in the future (in use or novel). Although graphically, raster plots and activity histograms show the differences between control neuronal activity and enhanced pathological activity observed in DA-depleted circuits [3–13], and the later, with activity seen after addition of L-DOPA, we show that cumulative activity and cumulative recruitment of neurons offer quantitative measurements and ranges capable of allowing concentration-response plots in future studies. L-DOPA significantly reduced the enhanced pathological activity of the DA-depleted circuit. In addition, we show that L-DOPA actions can be readily reversed upon washing off [13]. Therefore, the use of this preparation may also be used to shed light on the development of L-DOPA induced receptor hypersensitivity and later dyskinesia in animal models [19, 21]. Moreover, the combination of selective dopamine receptor ligands with transgenic mice would allow the testing of the different models of Parkinsonism [12, 14, 18, 19].

 In addition to enhanced activity in the DA-depleted microcircuit, the capability of having single-cell resolution allows to see the formation of coactive neuron sets or network states [20, 22, 23]. The analysis of network states shows the similarity between peaks of synchronization and shows that microcircuit dynamics (with LLE) is basically dependent on the recurrence of the same dominant network state that absorbs most active neurons [3]. This dynamics interrupts normal alternation between network states as occurring in active control microcircuits [20, 22, 23] and may be the cellular correlate of the increased oscillatory activity found in Parkinsonian subjects [8, 9, 11–13]. Interestingly, L-DOPA was capable of dissolving this dominant state in this stage of the disease and returning neuronal activity to control levels. In contrast, a D_1_-like receptor agonist could dissolve the dominant state but did not reduce enhanced pathological activity [3]. D_2_-like receptor agonists apparently decrease pathological activity [14] but do not avoid D_1_-like receptor hypersensitivity resulting in pathological states [19, 21]. A battery of D_2_-like receptor agonists may need to be tested with the present technique. Apparently, combinations of both agonists [18, 20, 24] with coadjutant agonists from other receptors present in striatal neurons may allow an increased understanding of anti-Parkinsonian therapeutics.

## 4. Conclusions

 Calcium imaging of populations of striatal neurons with single-cell resolution allows a clear and quantitative observation of anti-Parkinsonian actions of L-DOPA in DA-depleted striatal circuit recorded *in vitro*, paving the way for future bioassays for drug testing and correlations with disease stage, and even perhaps the achievement of functional biopsies. To reach these goals, the present preparation needs to be compared and correlated with known behavioral bioassays and in different stages of the disease, from early Parkinsonism to dyskinesias and wearing off.

 We have previously shown that in other basal ganglia nuclei it is enough to add antagonists for both D_1_- and D_2_-like receptors to the bath saline to induce the oscillatory neuronal activity [25] characteristic of Parkinson's disease. This is harder to see in the striatum, where several neurons need to be synchronized [11–13]. The present preparation, in combination with transgenic animals and other techniques, may allow the study of the extrinsic and intrinsic components that induce pathological activity.

 Finally, the present preparation may not only serve to study Parkinson's disease. Correlates between neuronal activity recorded *in vitro *and multi-unit/field recordings done *in vivo* may be correlated at the circuit level in many other nuclei and disease states.
